# Artificial Intelligence and Machine Learning in Diagnostic Radiology: A Paradigm Shift Toward Predictive Neuroimaging and Early Detection of Brain Disorders

**DOI:** 10.7759/cureus.107432

**Published:** 2026-04-20

**Authors:** Shubham Gupta, Narendra Kumar Arya, Vishwa Reddy, Rautesh Kumar Sharma, Satyanarayana Kummari, Ashish Kumar Shukla, Aashish Patel

**Affiliations:** 1 Department of Radiodiagnosis, Jammu University, Jammu, IND; 2 Department of Pediatrics, Guru Govind Singh Indraprastha University, Delhi, IND; 3 Department of Emergency Medicine, Apollo Medical College, Apollo Institute of Medical Sciences and Research, Hyderabad, IND; 4 Department of Radiology, Indian Railways (Northern Railway Divisional Hospital), Lucknow, IND; 5 Department of Radiology, All India Institute of Medical Sciences, Bibinagar, Hyderabad, IND; 6 Department of Radiodiagnosis, Santosh Deemed to Be University, Ghaziabad, IND; 7 Department of Internal Medicine, KAHER’s Shri BMK Ayurveda Mahavidyalaya, Shahapur, IND

**Keywords:** artificial intelligence, diagnostic radiology, machine learning, neuroimaging, predictive imaging

## Abstract

Artificial intelligence (AI) and machine learning (ML) are increasingly reshaping diagnostic radiology, particularly neuroimaging, by enabling a transition from traditional descriptive interpretation to predictive, quantitative, and precision-oriented analysis. The rising global burden of neurological and neuropsychiatric disorders, coupled with the exponential growth in imaging data complexity, has exposed the limitations of conventional, human-centered radiological assessment. This descriptive review synthesizes recent advances in AI- and ML-driven neuroimaging, with emphasis on their role in early disease detection, risk prediction, and clinical decision support. Key applications across major imaging modalities, including magnetic resonance imaging (MRI), computed tomography, positron emission tomography, functional MRI, and diffusion tensor imaging, are examined, encompassing brain tumor characterization, neurodegenerative disorders, stroke, epilepsy, and psychiatric and neurodevelopmental conditions. In addition to diagnostic performance, the review highlights AI-enabled workflow optimization and addresses critical challenges related to data heterogeneity, external validation, model interpretability, regulatory oversight, and ethical considerations. Although AI-driven approaches demonstrate substantial potential to enhance diagnostic accuracy, efficiency, and personalized patient care, their routine clinical integration remains limited by methodological and translational barriers. Overcoming these challenges through robust multicenter validation, development of explainable AI models, and sustained interdisciplinary collaboration will be essential to fully realize the promise of predictive neuroimaging and advance diagnostic radiology toward preventive and precision medicine.

## Introduction and background

Diagnostic radiology is a crucial component of contemporary clinical practice, enabling noninvasive visualization of anatomical and physiological processes on which the correct diagnosis, monitoring, and prognosis of disease are based [1]. In this area, neuroimaging is of particular clinical importance due to the brain's inherent complexity and the fact that neurological illness is often subtle and heterogeneous [2]. New imaging technologies, including magnetic resonance imaging (MRI), computed tomography (CT), positron emission tomography (PET), functional MRI (fMRI), and diffusion tensor imaging (DTI), have significantly improved the detection and characterization of brain pathology [3]. Nevertheless, the rising complexity and volume of imaging information have continued to burden traditional human-centered interpretative paradigms [4].

Neurological and neuropsychiatric conditions are a significant and increasing global health burden, and the burden is in part attributable to population aging, rising survival, and rising prevalence of disease [5]. Conditions such as Alzheimer’s disease (AD), Parkinson’s disease (PD), stroke, epilepsy, brain tumors, and major psychiatric disorders are significant contributors to long-term disability, reduced quality of life, and increased healthcare expenditure worldwide [6]. Notably, many neurological diseases have prolonged preclinical or prodromal phases during which subtle shifts in brain structure, function, or metabolism occur years before overt clinical symptoms manifest [7]. Early detection of disease gives a valuable opportunity to intervene in time, stratify risks, and achieve better outcomes [8].

**Figure 1:**
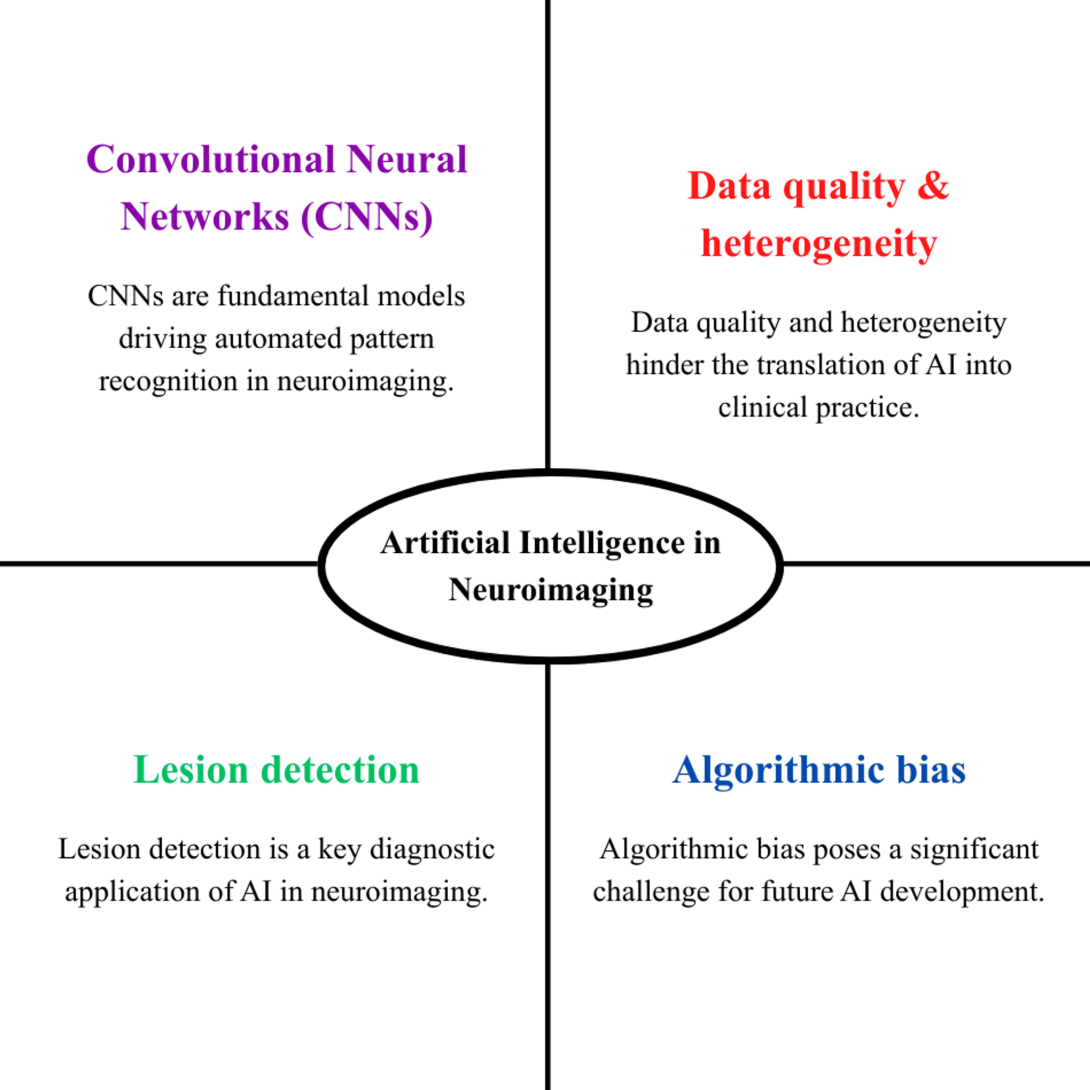
Core applications and challenges of AI in neuroimaging AI: artificial intelligence, CNNs: convolutional neural networks Image Credit: Authors using PowerPoint (Microsoft Corp., Redmond, WA, USA)

However, conventional neuroimaging analysis is often insensitive to these early and complex patterns, particularly when abnormalities are diffuse or subtle [9]. This limitation is not merely theoretical, as a previous study demonstrated diagnostic failure despite characteristic radiological features, with definitive diagnosis eluding conventional methods [9]. Such diagnostic failures underscore the need for computational tools capable of extracting clinically relevant information beyond human visual perception. Expert human judgment remains the cornerstone of radiological interpretation, yet studies report moderate inter-observer agreement (kappa 0.4-0.7) for many neuroimaging findings, with intra-observer variability also contributing to diagnostic inconsistency [10]. The accuracy of the diagnostic process can be influenced by various factors, including radiologists' experience, cognitive load, and fatigue, especially when assessing complex neuroimaging studies [11]. Furthermore, technological advances have led to high-dimensional imaging data comprising numerous sequences and thousands of images per scan [12]. Figure 1 provides a review of the key uses and issues of artificial intelligence (AI) in neuroimaging.

The increasing clinical workload on radiologists also reduces the time available to thoroughly examine images, which can lead to delays in the diagnostic process or an inability to detect issues [13]. Together, these issues underscore the importance of complex computational software capable of extracting clinically relevant information from large-scale neuroimaging data with greater consistency and efficiency [14]. Machine learning (ML) and AI have emerged as potent technologies that will help overcome these limitations [15]. AI is a concept applied to computational systems designed to perform actions that a human can help perform through their intelligence. In contrast, ML involves algorithms that can learn more complex patterns from data and improve at performing specific tasks [16].

Specifically, deep learning (DL), an advanced subset of ML, has emerged as a leading approach in automated medical image processing, demonstrating strong performance in analyzing complex imaging data [17]. These models automatically learn hierarchical feature representations, enabling the identification of subtle imaging signatures that may not be readily detectable by human visual assessment; consequently, they are particularly valuable in neuroimaging applications such as lesion detection, tissue segmentation, and disease classification [18]. AI in diagnostic radiology extends beyond task automation and increasingly supports data-driven clinical decision-making and workflow enhancement [19]. AI-enabled neuroimaging contributes to a shift toward predictive and preventive medicine, complementing traditional reactive, symptom-based diagnostic approaches [20].

The detection of imaging biomarkers associated with disease risk, progression, and treatment response enables earlier patient identification and supports more individualized clinical decision-making [21]. In conditions such as neurodegenerative diseases, cerebrovascular disorders, and brain tumors, predictive neuroimaging models have shown potential in improving early diagnosis, prognostication, and personalized treatment planning, supporting the broader goals of precision medicine [22].

Despite rapid technological advancements, the clinical implementation of AI and ML in diagnostic radiology remains in its early stages. Key challenges, including data quality variability, algorithmic bias, limited model interpretability, regulatory constraints, and lack of external validation, continue to restrict widespread adoption. Additionally, the existing literature is heterogeneous in methodology and clinical focus, necessitating critical synthesis. This review provides a structured, clinically oriented synthesis of current evidence, with a specific emphasis on predictive neuroimaging and early detection of brain disorders, and evaluates translational challenges and future directions for clinical integration.

Objectives of the review

This review aims to comprehensively examine current applications of AI and ML in diagnostic radiology, with a specific focus on neuroimaging. It evaluates the role of AI-based techniques in early diagnosis and prediction of brain disorders and assesses their impact on diagnostic accuracy, workflow efficiency, and clinical decision-making. Additionally, the review analyses key methodological limitations, ethical considerations, and barriers to clinical translation, offering a focused perspective on integrating AI into neuroimaging practice.

Methodology

This descriptive narrative review was conducted to consolidate and critically analyze the available literature on the use of AI and ML in diagnostic radiology, with specific attention to neuroimaging and early diagnosis of brain disorders. Given the rapidly evolving and heterogeneous nature of AI research in neuroimaging, a narrative approach was selected to enable flexible synthesis of diverse methodologies and clinical applications rather than strict quantitative aggregation. Unlike a systematic review, this study does not follow PRISMA guidelines and does not include a predefined protocol or quantitative study selection process. To gather both clinical and technical research articles published between January 2021 and August 2025, a comprehensive literature search was conducted across multiple electronic databases, including PubMed, Scopus, Web of Science, IEEE Xplore, and Google Scholar.

The chosen search strategy involved controlled-vocabulary and free-text keyword combinations, such as AI, ML, DL, diagnostic radiology, neuroimaging, brain disorders, early detection, and predictive imaging, refined with Boolean operators. An example search string used in PubMed was: (“artificial intelligence” OR “machine learning” OR “deep learning”) AND (“neuroimaging” OR “MRI” OR “CT” OR “brain imaging”) AND (“neurological disorders” OR “brain disorders” OR “early diagnosis”). Representative high-impact and clinically relevant studies were prioritized to ensure applicability to current clinical practice. Although the primary focus was on recent literature (2021-2025), selected earlier foundational studies were considered where necessary to provide conceptual context. Articles published in languages other than English and those published outside the specified time period were excluded, a limitation that may introduce language bias.

The initial search yielded 296 articles. After removing duplicate records and performing preliminary relevance screening, 112 articles were assessed in detail. Of these, 50 studies were ultimately included based on relevance and methodological quality. Articles were included if they were peer-reviewed original research studies or high-quality review articles on neuroimaging, neuroscience, or closely related clinical applications of AI in neurological disorders. They reported AI/ML applications in diagnostic, predictive, or prognostic contexts related to neurological or neuropsychiatric disorders. Articles such as case reports, editorials, opinion pieces, conference abstracts without full text, and studies involving only animal models were excluded. No formal study selection flow diagram was constructed, consistent with the narrative design of this review. Studies lacking sufficient methodological detail or clinical relevance were also excluded.

Relevant data were extracted based on study design, sample size, imaging modality, AI/ML techniques used, targeted brain disorder, and reported clinical outcomes. The extracted information was synthesized qualitatively to identify major themes, methodological trends, and research gaps. Although a formal risk-of-bias assessment tool was not used, the included studies were critically appraised based on study design, sample size, validation approach, and clinical applicability, with particular emphasis on external validation and the reporting of performance metrics. Where applicable, emphasis was placed on studies reporting quantitative performance metrics, external validation, and clinical applicability, while acknowledging that many included studies remain retrospective and heterogeneous in design.

Given the substantial heterogeneity in study designs, imaging modalities, patient populations, and outcome measures, quantitative pooling (meta-analysis) was not attempted. Accordingly, statistical measures such as pooled effect sizes, confidence intervals, or p-values are not reported, and findings are presented through qualitative synthesis. This qualitative synthesis provides an interpretative overview rather than a reproducible, systematic aggregation of evidence, consistent with the descriptive nature of the review.

## Review

Foundations of AI and ML in radiology

AI refers to computational systems designed to perform tasks that typically require human intelligence, such as learning, pattern recognition, and decision-making [23]. ML, a subset of AI, enables algorithms to learn from data and improve performance without explicit programming [16]. DL, a further subset of ML, employs artificial neural networks with multiple hidden layers to automatically learn hierarchical feature representations from large-scale, high-dimensional data [24]. These capabilities make AI particularly well-suited for analyzing complex medical images, where subtle patterns may not be readily detectable by human observers [18], thereby forming the foundation for many current and emerging neuroimaging applications.

Radiology ML methods are broadly classified into supervised and unsupervised learning approaches [25]. Supervised learning uses labeled datasets, in which input images are paired with known outputs such as diagnoses, segmentation masks, or clinical outcomes, enabling models to learn direct input-output relationships [17]. This approach underpins key radiological applications, including lesion detection, disease classification, and image segmentation [3]. In contrast, unsupervised learning analyses unlabeled data to identify inherent structures or patterns without predefined outputs [26]. Although less commonly used in routine clinical workflows, it is increasingly applied to characterize disease heterogeneity and identify novel imaging phenotypes in complex neurological conditions [9].

Convolutional neural networks (CNNs), a type of DL model specifically designed for image analysis, are the most widely used architecture in medical imaging [27]. CNNs process image data by learning spatial features directly from pixel values, enabling detection of patterns such as edges, textures, and anatomical structures [24]. Through hierarchical feature extraction, these models capture increasingly complex image characteristics, enabling the identification of subtle abnormalities that may be difficult to recognize visually [18]. CNNs have demonstrated strong performance in lesion localization, tissue segmentation, image classification, and anomaly detection, making them central to AI-based diagnostic radiology workflows [22].

**Figure 2:**
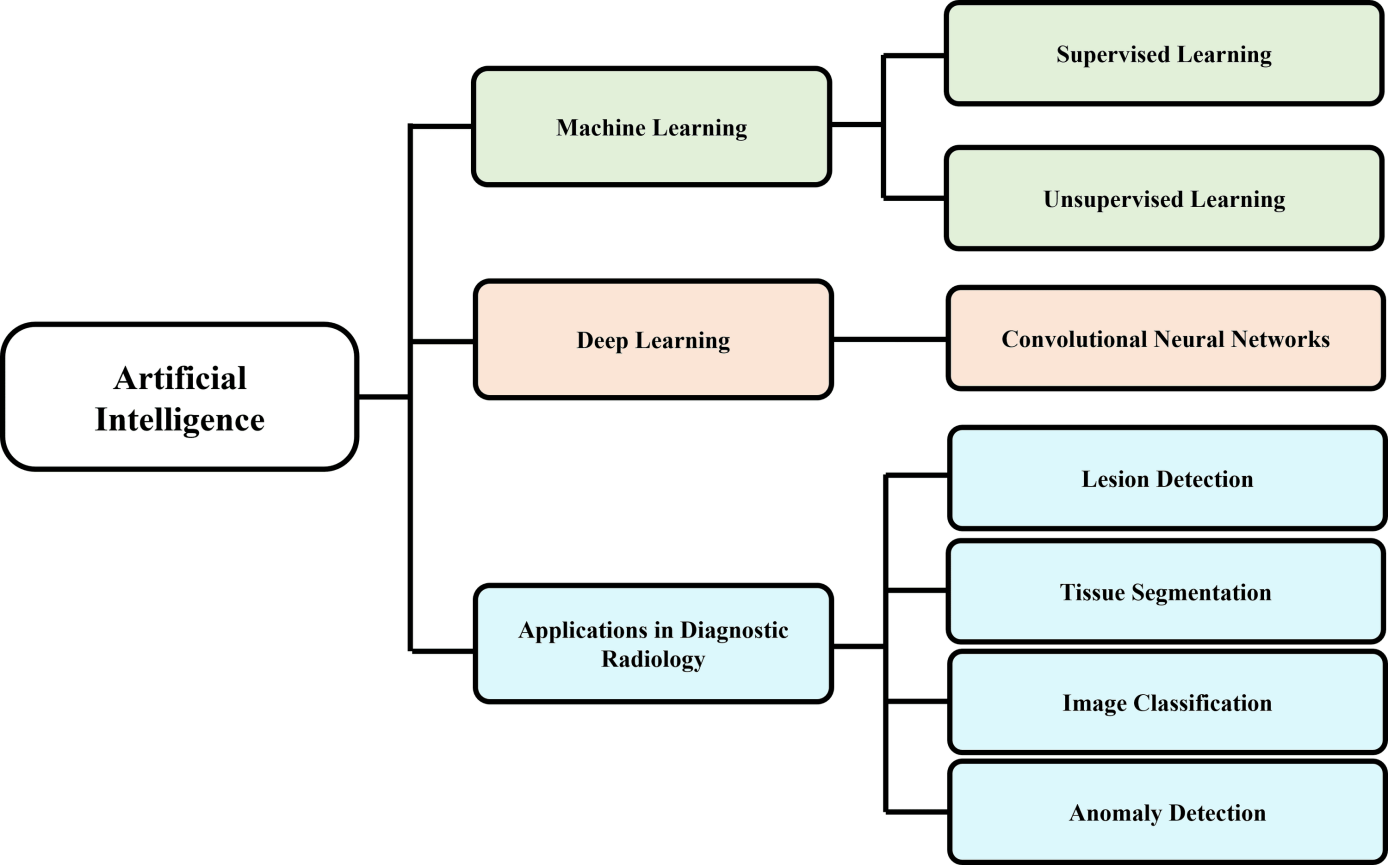
AI architectures and learning paradigms in diagnostic radiology AI: artificial intelligence Image Credit: Authors using PowerPoint (Microsoft Corp., Redmond, WA, USA)

Overall, these foundational AI methodologies enable a transition from qualitative visual interpretation to quantitative, data-driven analysis, underpinning subsequent applications across neurological disorders while also introducing challenges related to generalizability, interpretability, and clinical integration. Figure 2 illustrates the relationship between AI techniques and diagnostic radiology applications.

Neuroimaging modalities enhanced by AI

AI has enhanced multiple neuroimaging modalities by improving image quality, diagnostic accuracy, and quantitative analysis [28]. MRI remains the primary focus due to its superior soft-tissue contrast and ability to capture multidimensional aspects of brain structure and function [3]. AI-based reconstruction techniques enable faster MRI acquisition by generating high-quality images from undersampled data, thereby reducing scan time and improving patient comfort [29]. Additionally, DL-based denoising, motion correction, and super-resolution methods improve image fidelity without increasing acquisition burden [18].

In CT, AI has contributed to noise reduction and radiation dose optimization [30]. DL algorithms can generate diagnostically reliable images from low-dose CT scans, reducing radiation exposure while preserving clinically relevant information [16]. AI-assisted CT analysis is particularly valuable in acute neuroimaging settings, such as stroke and traumatic brain injury, where rapid detection of intracranial hemorrhage, ischemia, and mass effect supports timely clinical decision-making [31].

AI has also advanced functional and molecular imaging modalities, including PET and single-photon emission CT (SPECT) [32]. AI-driven reconstruction and attenuation correction improve the signal-to-noise ratio and quantitative resolution, thereby facilitating the detection of subtle metabolic abnormalities [24]. These improvements are particularly relevant in neurodegenerative diseases, where metabolic changes often precede structural alterations [7]. Similarly, AI-based analysis of fMRI and DTI enhances the evaluation of temporal dynamics and connectivity patterns, enabling more accurate mapping of functional networks and white matter pathways [33].

Collectively, these advancements demonstrate that AI enhances both image acquisition and post-processing across modalities, enabling a shift from qualitative interpretation to quantitative, data-driven analysis [34]. This cross-modality integration underpins predictive neuroimaging and translational research; however, variability in imaging protocols, scanner types, and data quality continues to affect model generalizability and clinical implementation [35].

AI in brain tumor detection and classification

Brain tumor imaging represents one of the most advanced and clinically relevant applications of AI in neuroimaging [36]. Conventional tumor assessment relies on expert interpretation of multiparametric MRI, which is time-consuming and subject to interobserver variability [10]. DL-based AI models have demonstrated high accuracy in tumor detection, segmentation, and classification in research settings, enabling more consistent and reproducible assessments; however, these findings largely derive from retrospective studies, and variability across datasets and validation approaches remains a key limitation [37].

CNNs play a central role in automated tumor segmentation within AI-assisted neuro-oncology workflows [17]. By integrating multiple MRI sequences, these models generate voxel-level segmentation maps that delineate tumor core, peritumoral edema, and necrotic regions [38]. Such quantitative segmentation supports surgical planning, radiotherapy targeting, and longitudinal treatment monitoring [21]. While automation reduces radiologist workload for repetitive tasks and enhances inter-institutional consistency, differences in imaging protocols and scanner variability may affect model performance across centers [13].

Beyond segmentation, AI has shown potential in tumor grading and molecular subtype prediction [39]. Using imaging characteristics from multiple modalities, AI models can differentiate low-grade from high-grade gliomas. Emerging research in radiogenomics suggests that imaging features may be used to predict molecular markers such as 1p/19q codeletion and isocitrate dehydrogenase mutation status; however, these approaches remain investigational and are not yet sufficiently validated for routine clinical use [24]. This noninvasive estimation of tumor biology may support precision oncology, particularly when tissue sampling is limited, although prospective validation and standardization are required before clinical adoption [22].

AI-based predictive modeling has also been applied to assess tumor progression and treatment response [40]. By analyzing longitudinal imaging data, AI systems can help distinguish true tumor progression from treatment-related effects such as pseudoprogression or radiation necrosis, a persistent challenge in neuro-oncology [31]. Predictive analytics may further help identify patients at higher risk of recurrence or poor outcomes, enabling more personalized surveillance strategies and treatment planning [35]. However, most of these models lack external validation and are trained on relatively homogeneous datasets, limiting their generalizability across diverse clinical populations.

Overall, AI applications in brain tumor imaging demonstrate substantial potential to enhance diagnostic accuracy and clinical decision-making; however, consistent with other domains of neuroimaging, challenges related to data heterogeneity, validation, and clinical integration remain significant barriers to routine implementation. Table 1 summarizes key applications and clinical utilities.

**Table 1:** AI applications in brain tumor imaging Compiled by the authors based on information derived from the referenced studies [2,13,21,24,27,31,35] AI: artificial intelligence, ML: machine learning, DL: deep learning, CNNs: convolutional neural networks, MRI: magnetic resonance imaging, PET: positron emission tomography

Application area	AI methods	Imaging modalities	Clinical utility	References
Tumor detection and segmentation	DL, CNNs	Multiparametric MRI	Automated delineation of tumor components with improved consistency	Xu et al., 2023 [27]
Quantitative tumor assessment	CNN-based segmentation	MRI	Volumetric analysis for surgical and radiotherapy planning	Valliani et al., 2019 [21]
Workflow optimization	Automated AI pipelines	MRI	Reduced radiologist workload and improved reporting efficiency	Onciul et al., 2025 [13]
Tumor grading	ML, DL	Multimodal MRI	Differentiation of low- and high-grade gliomas	Gutman et al., 2024 [24]
Molecular subtype prediction (radiogenomics)	ML/DL-based feature analysis	MRI ± PET	Noninvasive prediction of tumor molecular characteristics	Carvalho Macruz et al., 2024 [2]
Tumor progression assessment	Predictive modeling, DL	Longitudinal MRI	Differentiation of true progression from treatment effects	Șerban et al., 2025 [31]
Outcome and recurrence prediction	Predictive analytics	Imaging + clinical data	Early identification of high-risk patients	Verma et al., 2022 [35]

Predictive neuroimaging in neurodegenerative disorders

Neurodegenerative disorders are among the most promising applications of AI in neuroimaging, owing to their prolonged preclinical phases and progressive nature [5]. Diseases such as AD, PD, and Huntington’s disease (HD) are characterized by subtle structural and functional brain alterations that may occur years before clinical symptoms become evident [7]. Conventional neuroimaging plays an essential role in diagnosis and exclusion of other conditions, but often lacks sensitivity for detecting these early changes [9]. In this context, AI-driven predictive neuroimaging offers the potential to identify early biomarkers and model disease trajectories [20].

AI-based analysis of structural MRI has enabled the detection of subtle cortical and hippocampal atrophy in AD and mild cognitive impairment (MCI), which may not be readily identifiable through visual assessment alone [18]. DL models have achieved AUC values of 0.85-0.95 for distinguishing normal aging from MCI and early AD in research settings; however, external validation across diverse populations remains limited [24]. Similarly, AI-enhanced PET imaging improves detection and quantification of amyloid and tau pathology, supporting earlier disease stratification and staging [32]. Longitudinal predictive models further estimate the likelihood of progression from MCI to AD, enabling earlier intervention and patient counseling, although their clinical integration remains under evaluation [35].

AI-assisted neuroimaging has also been applied in PD to identify early alterations in the basal ganglia, substantia nigra, and associated motor and cognitive networks [14]. ML approaches that integrate multimodal MRI and functional imaging data have demonstrated the ability to differentiate idiopathic PD from atypical Parkinsonian syndromes and model disease progression [26]. In HD, which has a well-defined genetic basis, AI-based imaging enables detection of presymptomatic neurodegenerative changes in gene carriers, including alterations in cortical thickness, white matter integrity, and functional connectivity [30,22]. These imaging biomarkers also serve as potential objective endpoints in clinical trials.

Across neurodegenerative disorders, a key strength of AI lies in its ability to integrate high-dimensional imaging data with clinical and cognitive variables, facilitating early biomarker discovery and predictive modeling [40]. However, as in other domains, many studies rely on retrospective datasets with limited external validation, and variability in imaging protocols and population characteristics continues to constrain generalizability and routine clinical adoption.

AI applications in stroke diagnosis and prognostication

Stroke represents a time-critical neurological emergency in which rapid diagnosis and imaging interpretation are essential for optimal patient outcomes [12]. The application of AI in stroke imaging has expanded rapidly, driven by the need to accelerate diagnosis, support therapeutic decisions, and improve prognostication [28]. A key clinical application is the automated differentiation between ischemic and hemorrhagic stroke on CT and MRI, enabling faster triage and timely initiation of treatment [31].

DL algorithms have demonstrated sensitivity of 95-98% and specificity of 90-95% for intracranial hemorrhage detection on non-contrast CT in controlled research settings, with performance comparable to or exceeding human readers in selected studies; however, these findings are primarily based on retrospective datasets and require prospective real-world validation [17]. Early ischemic changes in brain parenchyma are often subtle and difficult to detect visually, particularly in the hyperacute phase, where AI-based tools have shown potential to improve detection sensitivity [23]. These capabilities are especially valuable in high-volume emergency settings, where rapid and accurate interpretation is critical [14].

Beyond detection, AI enables quantitative assessment of the infarct core and ischemic penumbra through ML-based perfusion imaging analysis, providing standardized estimates of irreversibly damaged and salvageable brain tissue [29,35]. These metrics are central to patient selection for reperfusion therapies, including intravenous thrombolysis and mechanical thrombectomy, particularly in extended treatment windows [20]. AI-assisted perfusion analysis may reduce inter-observer variability and improve consistency in clinical decision-making, although variability in imaging protocols and model generalizability remain limitations [10].

AI-based prognostic models further support stroke management by predicting functional outcomes, hemorrhagic transformation risk, and treatment response [33]. Integration of imaging with clinical and laboratory data enables more individualized prognostication and informs rehabilitation planning [25]. However, many of these models are developed on relatively small or homogeneous datasets, limiting their generalizability across diverse populations and healthcare settings.

Overall, AI applications in stroke imaging highlight a consistent pattern across neuroimaging domains: strong performance in controlled research environments, coupled with ongoing challenges in validation, generalizability, and clinical integration. Table 2 summarizes key AI applications and their clinical impact.

**Table 2:** AI applications in stroke imaging Compiled by the authors based on information derived from the referenced studies [17,20,23,29,31,33,35] AI: artificial intelligence, ML: machine learning, CT: computed tomography, MRI: magnetic resonance imaging, MR: magnetic resonance, DL: deep learning

Application area	AI approach	Imaging modality	Clinical impact	References
Stroke type differentiation	DL	CT, MRI	Rapid differentiation between ischemic and hemorrhagic stroke	Șerban et al., 2025 [31]
Intracranial hemorrhage detection	DL	Non-contrast CT	High-sensitivity hemorrhage detection in acute settings	Khalighi et al., 2024 [17]
Early ischemia detection	ML	CT, MRI	Identification of subtle hyperacute ischemic changes	Abedi et al., 2021 [23]
Infarct core estimation	ML-based perfusion analysis	CT/MR perfusion	Quantification of irreversibly damaged tissue	Chen et al., 2022 [29]
Ischemic penumbra assessment	Predictive modeling	CT/MR perfusion	Identification of salvageable brain tissue	Verma et al., 2022 [35]
Treatment selection	AI-assisted decision support	Perfusion imaging	Patient selection for reperfusion therapies	Evangelou et al., 2025 [20]
Prognosis prediction	Predictive analytics	Imaging + clinical data	Prediction of outcomes and hemorrhagic risk	Calderone et al., 2024 [33]

AI in epilepsy and seizure localization

Neuroimaging in epilepsy presents specific challenges, particularly in drug-resistant focal epilepsy, where structural abnormalities may be subtle or not visible on conventional imaging [21]. Accurate localization of the epileptogenic zone is critical for successful surgical intervention, yet standard radiological interpretation may fail to identify the underlying pathology [34]. In this context, AI has emerged as a complementary tool, improving lesion detection and seizure localization through advanced pattern recognition techniques [41].

ML applied to structural MRI has improved the detection of subtle epileptogenic lesions, with studies reporting increases in sensitivity from 30-40% with standard radiology to 70-80% with AI-assisted analysis for focal cortical dysplasia [27]. AI-based approaches analyze complex textural and morphometric features across the cortex, enabling more objective and reproducible lesion identification [38]. These methods are particularly valuable in MRI-negative epilepsy, where conventional imaging appears normal despite focal seizure onset [42].

Integration of neuroimaging with electrophysiological data further enhances AI-based seizure localization [30]. Multimodal frameworks combining MRI, functional imaging, and electroencephalography capture both structural and functional abnormalities within epileptogenic networks [43]. This integrative approach improves presurgical localization accuracy and supports more individualized surgical planning [24].

AI is also increasingly used to predict surgical outcomes. Predictive models that integrate imaging, electrophysiological, and clinical variables estimate the likelihood of post-surgical seizure freedom, aiding in the selection of appropriate candidates for surgery and informing clinical decision-making [44].

Overall, AI applications in epilepsy imaging demonstrate improved diagnostic sensitivity and support more precise surgical planning; however, consistent with other neuroimaging domains, many models rely on limited datasets and lack robust external validation, which restricts their generalizability and routine clinical use [40].

Psychiatric and neurodevelopmental disorders

Psychiatric and neurodevelopmental disorders represent one of the most challenging yet potentially transformative applications of AI in neuroimaging [45]. Unlike many neurological conditions, these disorders typically lack focal structural abnormalities and are characterized instead by diffuse, heterogeneous alterations in brain structure and function [9]. Disorders such as schizophrenia, autism spectrum disorder (ASD), and major depressive disorder impose a substantial global burden. Yet, diagnosis and treatment selection remain largely based on clinical assessment due to the absence of robust objective biomarkers [6]. In this context, AI-driven neuroimaging has the potential to identify reproducible imaging signatures that may enhance diagnostic precision, risk stratification, and personalized treatment approaches [20].

In schizophrenia, ML and DL models have been used to identify abnormalities in cortical thickness, white matter integrity, and large-scale brain connectivity using structural MRI, functional MRI, and diffusion imaging [46]. AI-based approaches can differentiate individuals with schizophrenia from healthy controls using multimodal imaging features and, in some studies, have predicted transition to psychosis in high-risk populations [31]. Functional connectivity analysis has been particularly informative, revealing distributed network-level disruptions associated with cognitive and behavioral symptoms, thereby supporting a shift from region-based to network-level models of psychiatric pathology [33,18].

ASD presents additional complexity due to marked neurobiological and phenotypic heterogeneity across developmental stages [47]. AI-based neuroimaging studies have analyzed patterns of cortical maturation, functional connectivity, and network organization over time [24]. Multimodal ML models show potential in distinguishing individuals with ASD from neurotypical controls and in identifying biologically relevant subgroups within the autism spectrum [48]. Despite these advances, clinical translation remains limited due to variability in study design and a lack of large-scale validation [35].

In major depressive disorder, AI-assisted neuroimaging has primarily focused on functional alterations in limbic, prefrontal, and default mode networks [49]. While structural changes are often subtle and inconsistent, ML approaches applied to functional imaging data have shown potential for distinguishing affected individuals from controls and predicting treatment response [22]. Emerging evidence also suggests that AI may help identify neurobiological subtypes of depression, addressing limitations of symptom-based diagnostic frameworks [50].

Overall, AI applications in psychiatric and neurodevelopmental disorders highlight a shift toward network-level and multimodal analyses; however, this domain is characterized by significant methodological heterogeneity, small sample sizes, and confounding factors, such as medication effects, that limit reproducibility and generalizability. Consequently, most findings remain investigational, and substantial work is required before reliable clinical implementation can be achieved [14]. Table 3 summarizes disorder-specific findings and their clinical implications.

**Table 3:** AI applications in psychiatric and neurodevelopmental disorders Compiled by the authors based on information derived from the referenced studies [22,31,48] AI: artificial intelligence, MRI: magnetic resonance imaging, fMRI: functional magnetic resonance imaging, DTI: diffusion tensor imaging, ASD: autism spectrum disorder

Disorder	Imaging modalities	AI methods	Key findings	Clinical implications	References
Schizophrenia	MRI, fMRI, DTI	ML, DL	Alterations in cortical thickness, white matter integrity, and network connectivity	Diagnostic support and prediction of psychosis risk	Șerban et al., 2025 [31]
ASD	MRI, fMRI	ML	Atypical cortical maturation and functional connectivity patterns	Subgroup stratification and personalized intervention support	Basha et al., 2026 [48]
Major depressive disorder	fMRI	ML	Functional alterations in limbic, prefrontal, and default mode networks	Patient stratification and treatment response prediction	Sadr et al., 2025 [22]

Workflow optimization and decision support systems

Beyond diagnostic and predictive applications, AI plays a critical role in optimizing radiology workflows and improving clinical efficiency [41]. The increasing volume and complexity of neuroimaging studies have significantly increased radiologists' workload, contributing to potential diagnostic delays and errors [23]. AI-based workflow solutions address these challenges by automating repetitive tasks, prioritizing urgent cases, and providing clinical decision support [35].

Automated triage systems are among the most impactful AI applications in diagnostic radiology [28]. These systems can identify critical findings, such as intracranial hemorrhage, large vessel occlusion, or significant mass effect, and prioritize them for immediate review, thereby improving reporting turnaround times in emergency and high-throughput settings [31,45].

AI further enhances workflow efficiency by automating segmentation, quantitative analysis, and structured reporting [24]. Tasks that are traditionally labor-intensive, including volumetric measurements, lesion tracking, and longitudinal comparisons, can be performed rapidly and consistently using AI tools [38]. This enables radiologists to focus on higher-level interpretative and consultative roles, rather than routine manual processes [20].

Importantly, AI in radiology serves as a clinical decision-support tool rather than a replacement for human expertise [49]. Final interpretation and clinical responsibility remain with the radiologist, while AI contributes quantitative insights and highlights potential abnormalities [10]. Effective human-AI collaboration, particularly when systems are transparent and well-integrated into clinical workflows, may improve diagnostic accuracy, reduce cognitive load, and enhance professional efficiency [50].

Validation, generalizability, and clinical translation

Despite rapid technological progress, the clinical adoption of AI-based neuroimaging tools remains limited [19]. A major barrier is the lack of external validation, as many models are developed and tested on single-institution datasets [14]. These models often exhibit domain shift when applied to data from different institutions, scanner manufacturers, or patient populations, resulting in reduced performance and limiting clinical applicability [27]. Variability in imaging protocols, hardware, and demographic characteristics further constrains generalizability [9].

Dataset bias represents an additional challenge, including underrepresentation of certain demographic groups, disease subtypes, or imaging protocols, which may lead to systematic performance disparities and perpetuate healthcare inequalities if unaddressed [30,6]. Addressing these issues requires multicenter and multinational collaboration, the incorporation of diverse datasets, and the use of standardized benchmarking frameworks to improve transparency and reproducibility [35].

Regulatory approval and clinical validation further complicate translation into practice [22]. AI-based imaging tools must demonstrate not only technical accuracy but also measurable clinical benefit and safety [18]. Prospective validation studies and randomized clinical trials are increasingly necessary to establish their impact on diagnostic accuracy, workflow efficiency, and patient outcomes [33]. Additionally, continuously learning (adaptive) algorithms present regulatory challenges, necessitating ongoing monitoring and updated governance frameworks [40].

Successful clinical implementation also depends on integration with existing healthcare infrastructure, including picture archiving and communication systems and electronic health records [24]. Key determinants of adoption include user-centered design, interoperability, and clinician trust [10]. Even high-performing AI models may fail to achieve clinical impact if these practical considerations are not adequately addressed [28].

**Figure 3:**
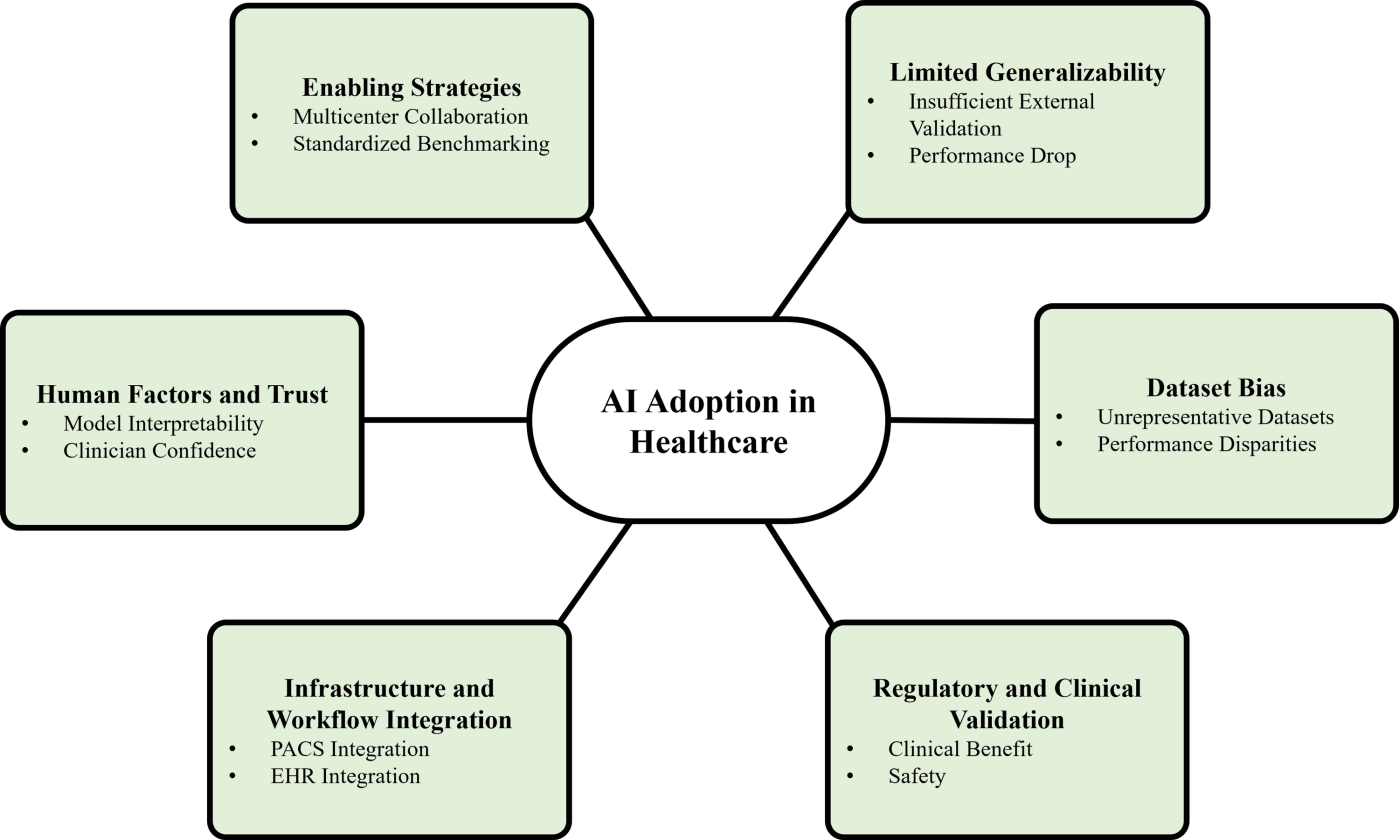
Barriers and enablers of AI adoption in healthcare AI: artificial intelligence, PACS: Picture Archiving and Communication System, EHR: Electronic Health Record Image Credit: Authors using PowerPoint (Microsoft Corp., Redmond, WA, USA)

Collectively, these challenges highlight a recurring theme across AI applications in neuroimaging: while technological capabilities are advancing rapidly, limitations related to validation, bias, and system integration continue to constrain routine clinical adoption. Figure 3 summarizes the key barriers to implementation.

Ethical, legal, and data privacy considerations

The integration of AI into diagnostic radiology raises important ethical, legal, and data privacy considerations that must be addressed alongside technological advancement [42]. Interpretability remains a major challenge, as many DL systems function as “black boxes,” offering limited insight into the features or mechanisms that drive their predictions, a barrier to clinician trust and regulatory approval [17]. This lack of explainability can complicate decision-making in high-stakes clinical contexts and limit acceptance in routine practice [10].

In addition to interpretability, multiple forms of bias, including selection bias, algorithmic bias, and representation bias, pose significant risks. Unrepresentative datasets may result in systematic performance disparities across demographic groups or disease subtypes, potentially reinforcing existing healthcare inequalities [6,30]. Addressing these issues requires inclusion of diverse populations during model development, continuous performance monitoring, and transparent reporting of limitations [35].

Legal accountability in AI-assisted diagnosis remains unclear, with ongoing debate regarding whether responsibility lies with clinicians, healthcare institutions, or technology developers. This uncertainty underscores the need for well-defined legal frameworks and professional guidelines that clearly establish roles, responsibilities, and standards of care within AI-integrated workflows [49].

Regulatory oversight is critical for safe clinical implementation. AI-based imaging tools must meet evolving regulatory standards that evaluate not only technical performance but also clinical safety, effectiveness, and real-world impact [22]. Adaptive algorithms that continuously learn from new data introduce additional regulatory challenges, requiring ongoing validation and post-deployment monitoring [40].

Data privacy and security are central to responsible AI implementation in neuroimaging. The use of large-scale patient data raises concerns related to confidentiality, data ownership, and informed consent [24]. Robust data governance frameworks, anonymization protocols, and compliance with data protection regulations are essential for maintaining public trust, alongside transparent communication with patients about the use of AI in clinical care [18].

Collectively, these considerations highlight that ethical, legal, and regulatory challenges are not isolated issues but interconnected factors that influence the safe and equitable adoption of AI in neuroimaging. A framework grounded in transparency, accountability, fairness, and patient-centered governance is essential to ensure sustainable and responsible integration into clinical practice [28,50].

Limitations and future directions

Despite substantial progress, the clinical implementation of AI in diagnostic radiology remains constrained by several key limitations. A major challenge is the limited availability of large, high-quality, and well-annotated neuroimaging datasets required for robust model development and external validation. Model generalizability is further compromised by heterogeneity in imaging protocols, scanner platforms, and patient populations across institutions.

The lack of interpretability in many DL models continues to limit clinicians' trust and accountability, particularly in high-stakes diagnostic scenarios. Additionally, the predominance of retrospective study designs and the scarcity of prospective, real-world validation studies restrict confidence in reproducibility and long-term clinical impact.

Future research should prioritize developing transparent and explainable AI models to enhance clinical trust and facilitate responsible adoption. Integration of multimodal data, including imaging, clinical, and molecular information, offers significant potential to advance precision neuroimaging and personalized care. Large-scale, multicenter studies are essential to establish clinical utility, safety, and generalizability across diverse populations.

Furthermore, sustained collaboration among clinicians, data scientists, and regulatory bodies will be necessary to standardize evaluation frameworks, address ethical and legal challenges, and ensure effective long-term integration of AI technologies into routine radiological practice. Overall, future progress will depend not only on technological innovation but also on addressing systemic barriers to clinical translation.

## Conclusions

AI and ML are augmenting diagnostic radiology, enabling a transition toward more quantitative and predictive neuroimaging while complementing rather than replacing conventional human interpretation. The range of neurological applications of high-potential AI-based systems has spanned the early detection of neurodegenerative disorders, state-of-the-art brain tumor characterization, stroke diagnosis, seizure localization in epilepsy, and the discovery of novel biomarkers in psychiatric and neurodevelopmental disorders, as described in this review. In addition to improving the quality of the diagnostic process, AI is highly useful for workflow optimization and clinical decision-making, thus confirming its status as an augmentative technology that enhances radiologists' experience rather than replacing it. However, the application of these advances in a clinical setting has been contingent on resolving significant challenges involving data heterogeneity, external validation, model interpretability, regulatory approval, and ethical governance. Strong multicenter, prospective studies; the development of transparent, comprehensible AI models; and long-term collaboration among clinicians, data scientists, and regulators will be the next steps. With responsible integration, AI-driven neuroimaging has the potential to enable early disease detection, support personalized care, and improve patient outcomes, advancing diagnostic radiology within predictive and preventive medicine.
